# Constantly marching forward…

**DOI:** 10.4103/0301-4738.60067

**Published:** 2010

**Authors:** Barun Kumar Nayak

**Affiliations:** P. D. Hinduja National Hospital and Medical Research Centre, Veer Savarkar Marg, Mahim, Mumbai - 400 016, India. E-mail: editor@ijo.in

The time has come again to review the progress of the Indian Journal of Ophthalmology (IJO) since its previous report published a year ago.[1] In the past year the entire editorial team has contributed significantly in maintaining the high standard attained earlier.[2] We published all the six issues on time without compromising on the quality. A total of 140 articles were published in the year 2009.

**Figure 1:**
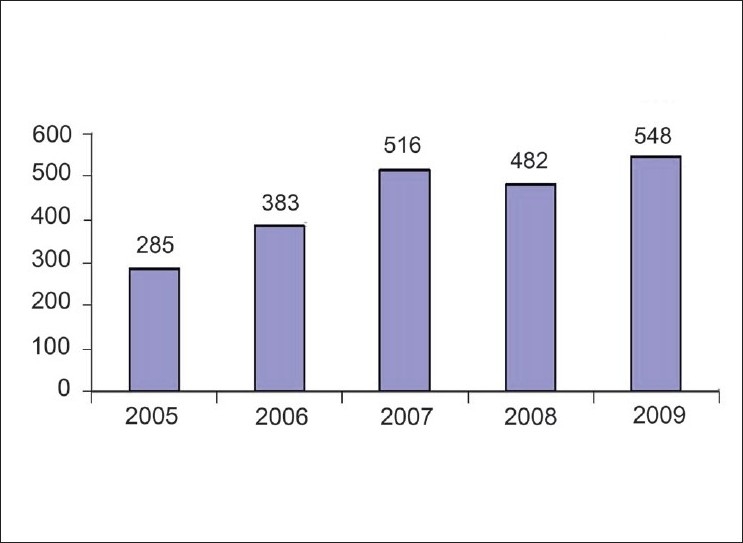
Year-wise submissions of manuscripts to IJO

Five hundred and forty-eight manuscripts were submitted in 2009, which was much higher than the previous year [Fig. 1]. Of the articles on which the decision was taken the same year, 27% were accepted. A total of 1776 authors have registered on our website and 386 out of these have submitted manuscripts in 2009, with 37 of them submitting more than one article. Of the total submissions, 192 (35%) manuscripts have been submitted from abroad, which was 32% in 2008. The number of original articles submitted from abroad has increased to 80 from the previous year's figure. of 75.

**Figure 2:**
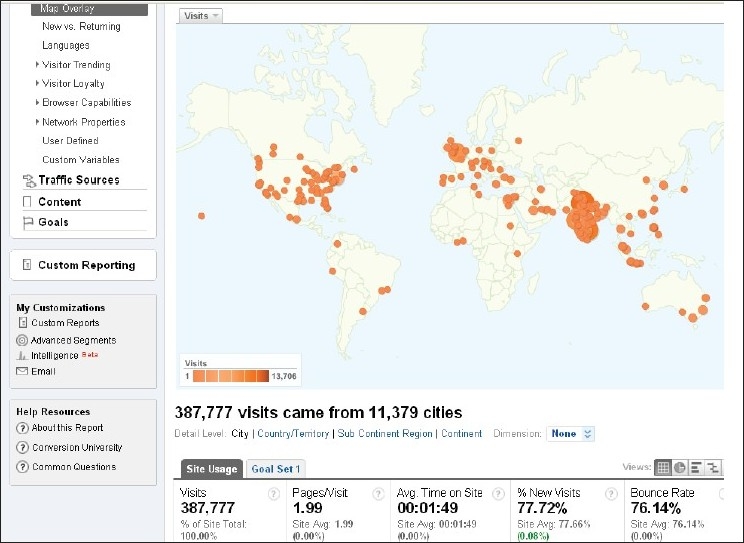
World map depicting places from where people are accessing the website

**Figure 3:**
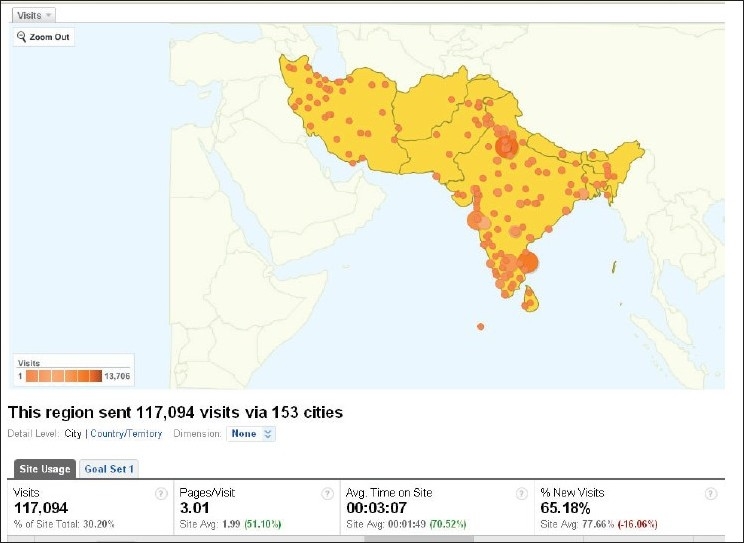
About one third visits on our website came from these cities

Our website <www.ijo.in>, which adopts the policy of Open Access, is getting even more popular, and is accessed from almost every part of the world [Figs. 2 and 3].

**Figure 4:**
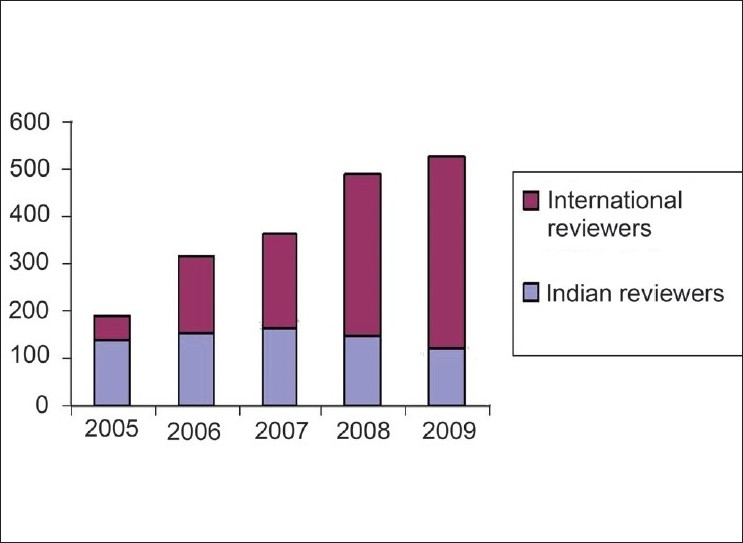
Year-wise break up of IJO reviewers

**Figure 5:**
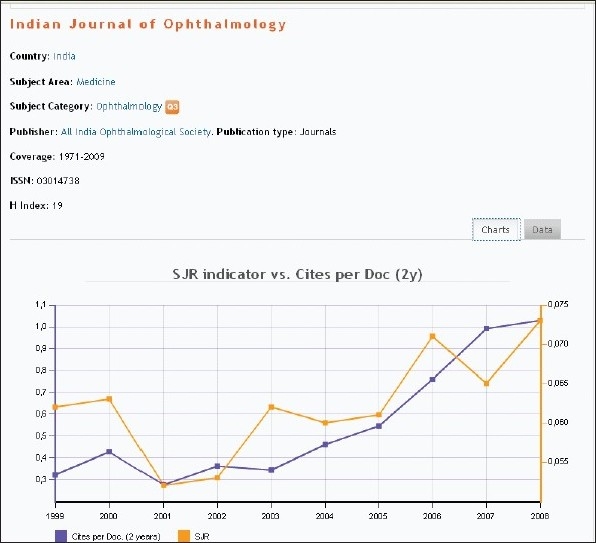
This graph shows SCImago Journal Rank (SJR) indicator and cites perDoc (2years)

**Figure 6:**
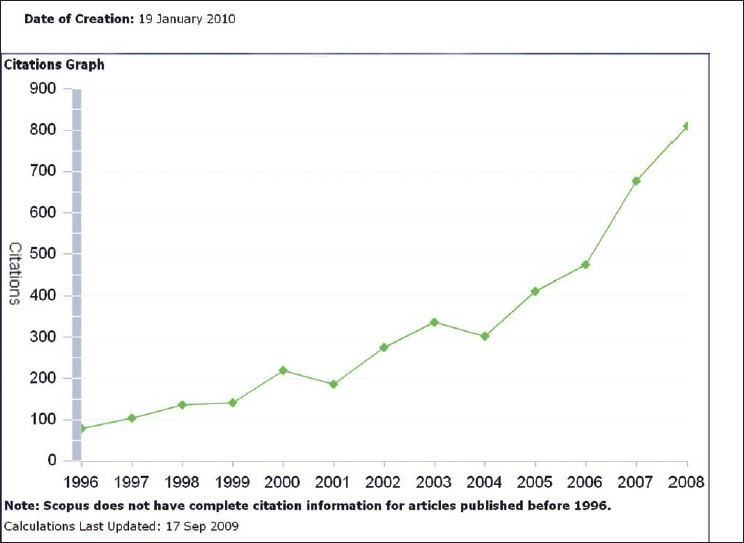
The citations graph shows the number of citations received by a journal in a given year

We are fortunate to have established a wide base of reviewers, the majority of whom are from abroad and experts in their respective fields. This rising trend in the number of reviewers [Fig. 4] has helped us to maintain our standard and improve our performance. The Open Access policy and our inclusion in PubMed Central along with more than 20 other indexing systems, have helped in constantly maintaining the rising trend of citations of articles published in IJO [Figs. 5 and 6].

Although uploading of full text of old journals on our website is complete for all the available issues of IJO, it is disheartening to note that eight issues could not be uploaded due to unavailability of their hard copies (1985/Volume 33/Issue 6, 1986/Volume 34/Issue 2/3/4/6, and 1987/Volume 35/Issue 5/6). I seek the help of all members in tracing these eight issues to complete the missing links of IJO.

The editorial office is doing everything in its capacity to help improve the standard of research and publications. The last research methodology workshop conducted in March 2009 was a great success. These workshops have become a regular feature of IJO in the past five years and have now culminated into a three part training series. Another initiative taken by the IJO to facilitate research was to provide the full text of any paid article to the members of the All India Ophthalmological Society (AIOS). This was made use of by a good number of AIOS members wherein 142 articles were requested for in a year.

The introduction of the “AIOS –IJO award” in the year 2009 was another step taken by IJO to attract good research papers from AIOS members. After a rigorous evaluation process six papers were chosen in the various categories. The list of awardees has been published in this issue of IJO. Furthermore, we are working on a project along with a management school on ‘How to attract good research papers for IJO’, the outcome of which, I am very confident, will help us in improving the quality of published papers in IJO.

Every journal is judged by its ‘Impact Factor’.[3] This year will be a testing ground for IJO as the first official ‘Impact Factor’ for the year 2010 will be published in the Journal of Citation Report in the year 2011. We are hoping to have an ‘Impact Factor’ close to one. This will be a great achievement for not only the editorial office and committee members, but also for all those authors and reviewers who have contributed towards this small success story called “IJO”.
